# Endplate Trauma During Implant Insertion Affects the Expulsion Risk of Anterior Lumbar Interbody Fusion Devices

**DOI:** 10.7759/cureus.36845

**Published:** 2023-03-29

**Authors:** Annette Kienle, Nicolas Graf, Youping Tao, Frank Heuer

**Affiliations:** 1 Medical Device Testing, SpineServ GmbH & Co. KG, Ulm, DEU; 2 Spine Surgery, SpineServ GmbH & Co. KG, Ulm, DEU; 3 Orthopedics, Ortho HUB Ventures UG, Filderstadt, DEU

**Keywords:** stand-alone cages, alif, biomechanics, mechanical test, endplate gouging, expulsion, failure, implant migration, anterior lumbar interbody fusion

## Abstract

Background

Anterior cage migration in anterior lumbar interbody fusion is a serious complication. To address this risk, cage designs are now available with integrated screw or blade fixation or specially designed surface geometries with large teeth or ridges. However, the implantation technique itself has not yet been addressed as a potential risk factor for cage migration. This study aimed to investigate whether a cage that is implantable without gouging the vertebral endplates has improved resistance to anterior migration.

Methodology

A novel three-piece modular cage was inserted between two vertebral body replacements (polyurethane (PU) foam grade 15 pcf) in two ways. In group 1 (modular), the cage was inserted in a wedge within a wedge fashion according to the manufacturer’s instructions such that damage to the PU foam was minimized. In group 2 (mono-bloc), the modular cage was inserted pre-assembled as a one-piece, mono-bloc device. This insertion method required impaction and increased the potential of gouging the PU surfaces. Then, an axial preload was applied to the PU test blocks to simulate the preload on the spine in vivo and an anteriorly direct expulsion force was applied to the cages.

Results

The mean expulsion yield load in the test group with modular implantation was 392 ± 19 N compared to 287 ± 16 N in the test group where the mono-bloc implants were inserted and endplate gouging occurred. This difference was statistically significant (p < 0.05). Thus, the onset of cage migration occurred at significantly higher loads in the test group with modular insertion without endplate gouging compared to one-piece impaction with gouging taking place. In contrast, the stiffness and the ultimate load were similar in both test groups (p > 0.05).

Conclusions

This study showed that the cage insertion technique may have a significant effect on the cage migration risk. Prevention of endplate gouging during cage implantation has the potential to improve the primary stability of the cage.

## Introduction

Anterior lumbar interbody fusion (ALIF) is an established technique for the treatment of various lumbar spinal diseases [1]. Several advantages of the ALIF technique have been previously documented, such as the restoration of foraminal height and sagittal spinal balance [2-4]. However, implant-related complications have also been reported [5-7], such as cage subsidence, cage migration, and even expulsion [8].

The biomechanical behavior of anterior interbody fusion cages is influenced by several factors. For example, it has been reported that cages with sharp teeth design have greater pull-out forces [9]. In an effort to optimize and evaluate the stability of ALIF cages, several studies have described the biomechanical results of different ALIF cage designs [9-12]. However, the detailed features of the resistance against static expulsion of ALIF cages are not yet known [13]. Moreover, it is still not well understood how the cage insertion technique may influence the risk of migration.

It is believed that a fundamental understanding of the biomechanical characteristics of implants is critical because the knowledge can be used as an important basis for future investigations, such as new implant designs. Additionally, the design and evaluation of new spinal implants is still an area of active and interesting research.

Therefore, this study aimed to investigate whether a novel, modular cage whose insertion method is aimed at reduced trauma to the endplates during insertion has a higher resistance to expulsion forces compared to a one-piece or mono-bloc cage where insertion is via simple impaction and gouging of endplate during insertion is inherent.

## Materials and methods

**Figure 1:**
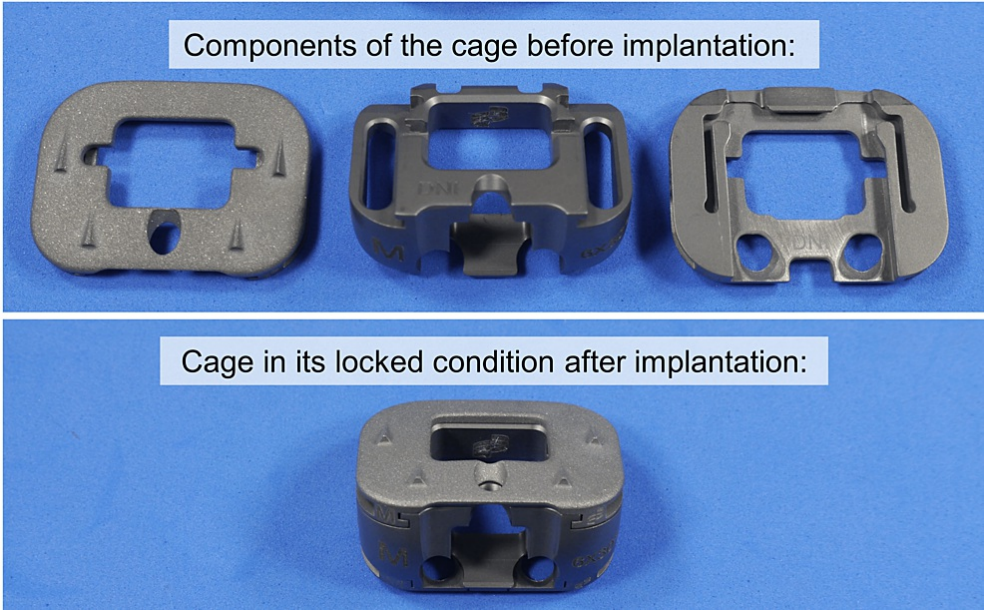
Novel modular anterior lumbar interbody fusion cage. Implantation is carried out with each of the two implant endplates (upper row, left, and right) and the implant body (upper row, middle) separately. After successful implantation, the implant body securely snaps into the endplates forming its final, locked mono-bloc condition (lower row).

In this study, a novel modular ALIF cage (Axis Spine Technologies Ltd., UK) was tested. This cage is composed of three parts, namely, an upper and a lower endplate and a wedged implant core. All parts are made of medical-grade titanium alloy (Ti6AL4V). The surface of the upper and lower implant endplates is serrated with teeth to provide immediate stability after implantation (Figure 1).

The modularity of this implant allows for stepwise insertion of the cage into the prepared disc. First, the two implant endplates are simultaneously inserted and positioned. Then, the implant core is pushed in between the endplates until it firmly snaps into place. The aim of this procedure is to avoid damage to the bony endplate during cage insertion while optimizing segmental distraction and lordotic angle.

Two test groups were defined, both with a sample size of five. In both test groups, the above modular cage was tested; however, the insertion technique differed between the groups. In the first test group, the cage was inserted according to the manufacturer’s instructions to minimize the impact of damage and gouging of the vertebral endplates (modular). In the second test group, the above cage was preassembled before testing and handled as if it was a mono-bloc or one-piece device. Mono-bloc cages are inserted via sequential impaction/hammering (mono-bloc).

Test blocks made of polyurethane (PU) foam (grade 15 pcf, ERP #1522-02, Sawbones Europe AB, Sweden) were manufactured with planar surfaces. The density of these blocks was chosen according to ASTM F2267 [14]. These blocks represented the adjacent vertebral bodies.

The PU test blocks with the implant in between were positioned in a custom-built pneumatically driven axial loading device. In this device, a constant compressive load (Fax) of 500 N was applied to both endplates via the planar test block surfaces. The amplitude of the axial compressive load was chosen according to ASTM F2077 [15].

The loading device was fixed on the base plate of a static material testing machine (Zwick/Roell, Type Z020) with a 20 kN load cell (GTM, Type xForce K, Class 0.5: F ≥ 0.5%) and the spindle as displacement transducer (accuracy <0.001 mm).

**Figure 2:**
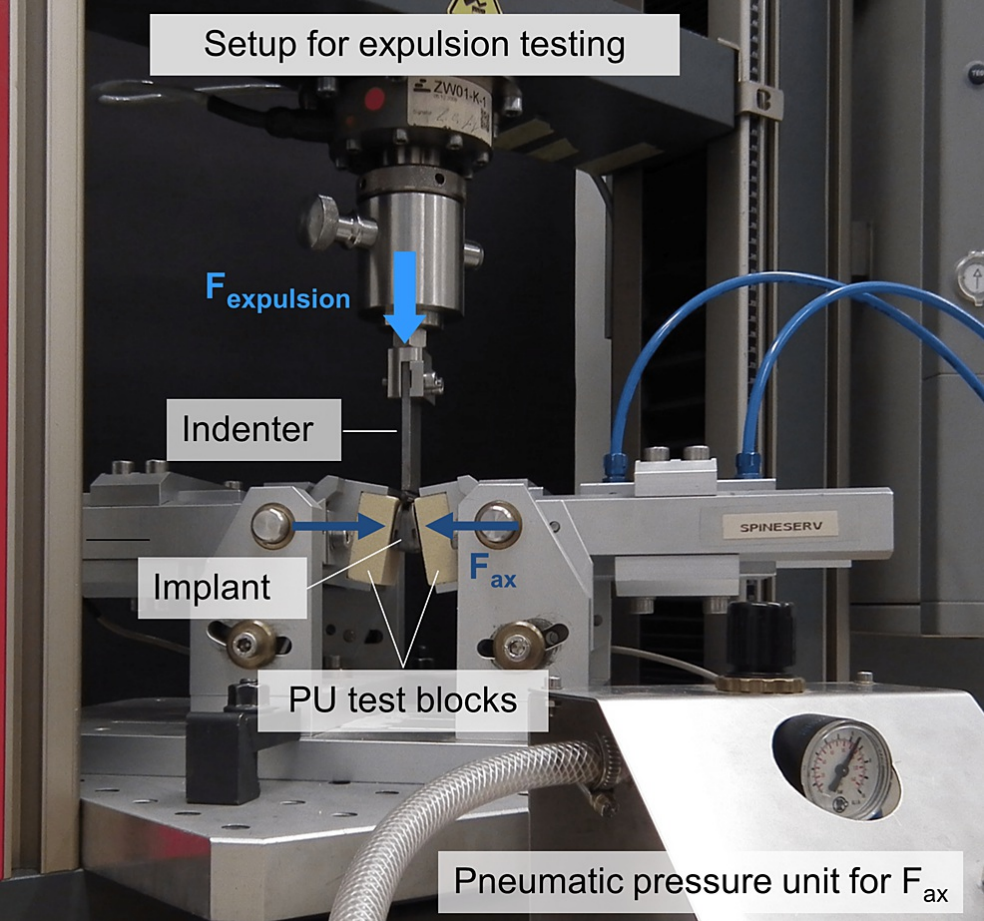
Test setup of the static expulsion test with the axial loading device mounted in the static material testing machine. The lordosis angle (20°) was considered by tilting the caudal and the cranial test blocks. F_expulsion_ = expulsion load; F_ax_ = constant axial preload.

**Figure 3:**
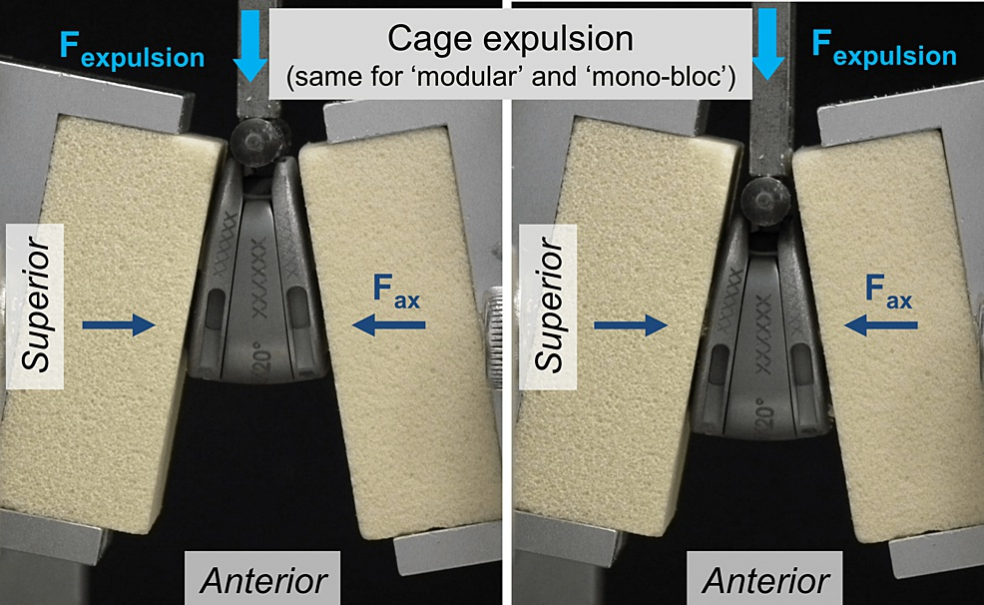
Test procedure of the static expulsion test. Left: start of testing; right: end of testing. F_expulsion_ = expulsion load; F_ax_ = constant axial preload

An indenter, which was connected to the force actuator of the testing machine, was used to apply an anteriorly directed expulsion force (F_exp_) to the cage at a constant rate of 5 mm/minute to expulse the cage from the space between the two test blocks (Figures 2, 3).

**Figure 4:**
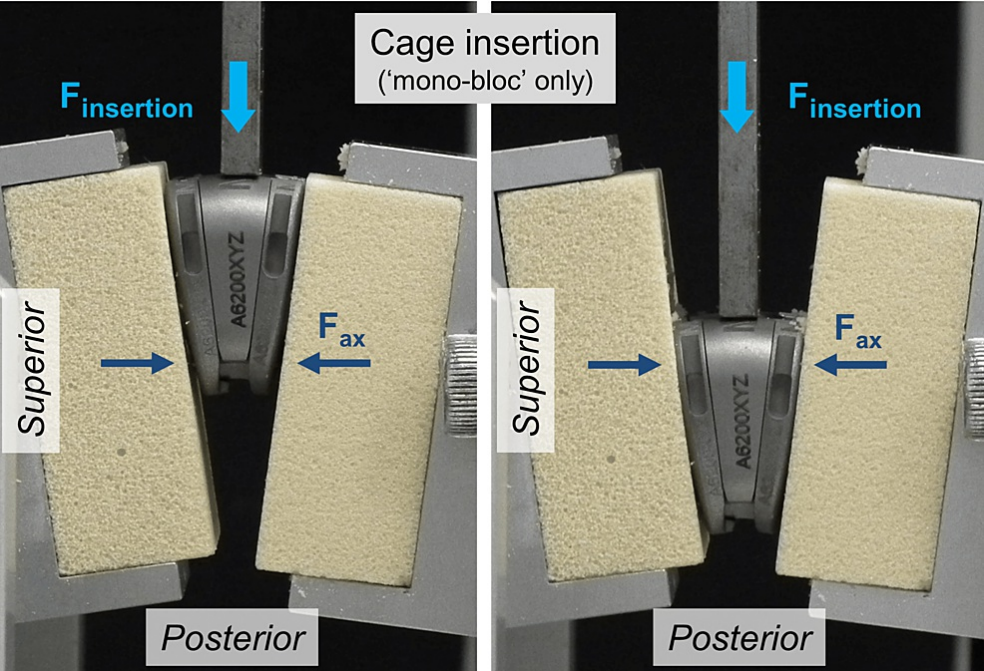
Test procedure of cage insertion in the mono-bloc test group. This insertion was not required in the modular group because there the implant components were inserted one after the other F_expulsion_ = expulsion load; F_ax_ = constant axial preload

The preassembled samples from the mono-bloc test group were inserted before testing using the same apparatus as described above. Only the test blocks were reversed and the load was applied in the posterior direction (Figure 4). Subsequently, the test blocks with the implant in between were again reversed and the expulsion test was performed.

Stiffness, yield load, and ultimate load were determined from the load-deformation curves (Table 1). A Student’s t-test was used with a significance level α of 0.05 to investigate whether the above target parameters significantly differed between the two test groups.

**Table 1:** Target parameters.

Parameter	Abbreviation	Units	Definition
Stiffness	S	N/mm	The slope of the initial, linear part of a load-displacement curve
Yield force	F_yield_	N	The load required to produce a permanent displacement equal to 0.1 mm
Ultimate expulsion force	F_ult_	N	The maximum load that can be applied to the implant

## Results

In the mono-bloc test group, the mean insertion force of the preassembled cages was 1,915 N.

**Figure 5:**
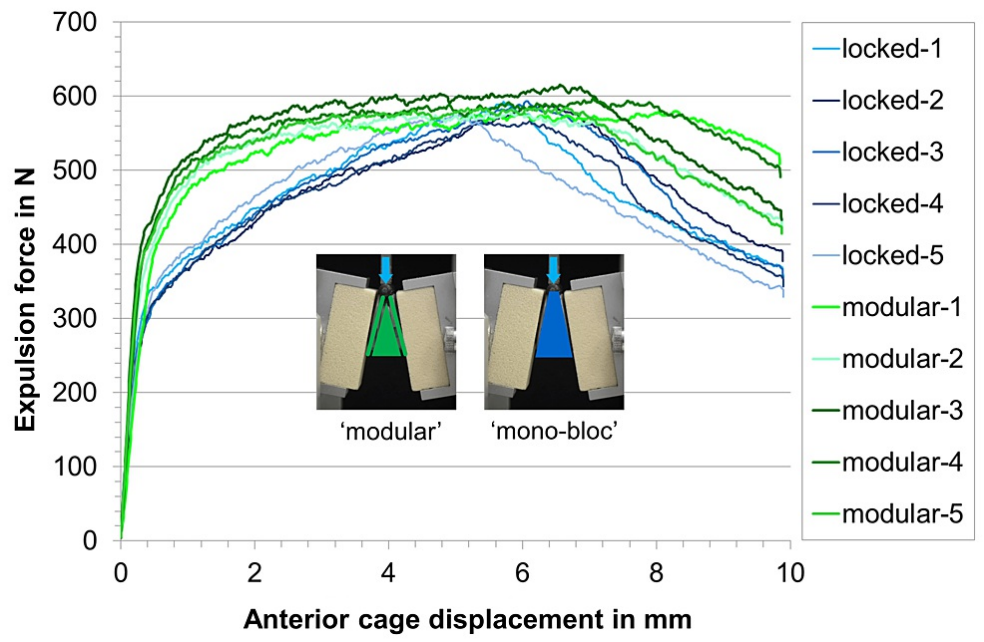
Individual load-displacement curves in the static expulsion test. In the modular group, the implant was stepwise inserted and assembled inside the disc space. In the mono-bloc group, the implant was preassembled and inserted as a whole into the simulated disc space.

**Figure 6:**
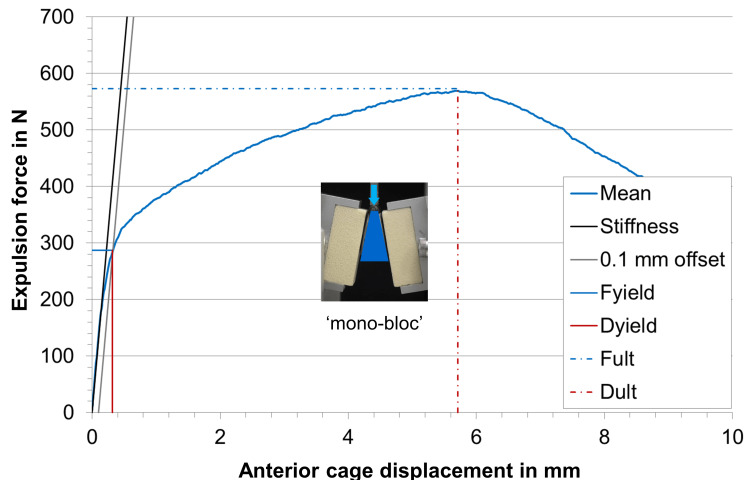
Mean load-displacement curve of the mono-bloc samples in the static expulsion test, calculated from all tested samples with stiffness, 0.1 mm offset displacement, yield load (Fyield), deformation at yield load (Dyield), ultimate load (Fult), and deformation at ultimate load (Dult).

**Figure 7:**
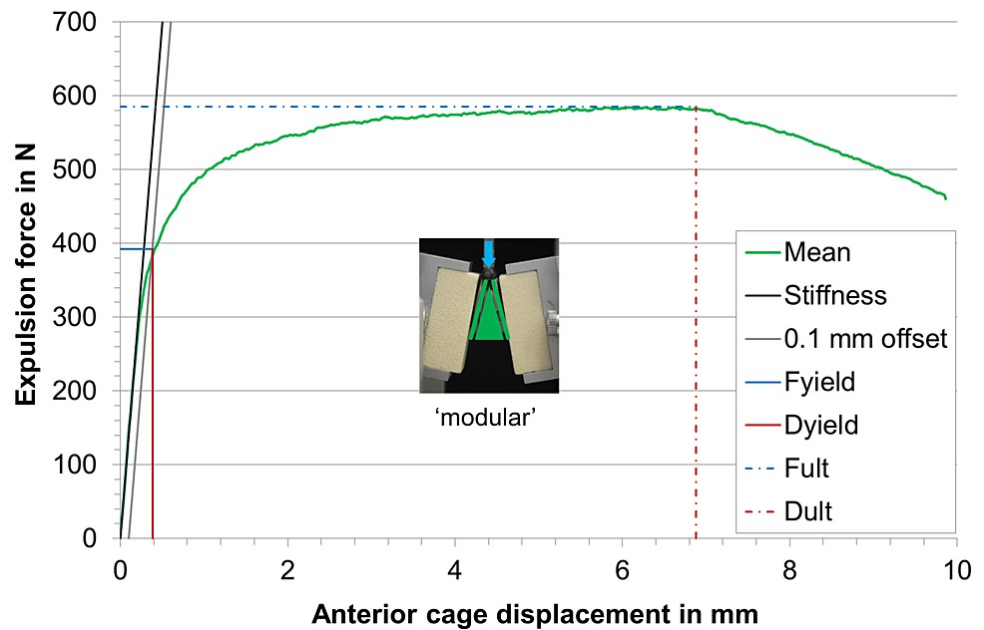
Mean load-displacement curve of the modular samples in the static expulsion test, calculated from all tested samples with stiffness, 0.1 mm offset displacement, yield load (Fyield), deformation at yield load (Dyield), ultimate load (Fult), and deformation at ultimate load (Dult).

Under static expulsion loading, the mean yield load (F_yield_) in the test series with modular implantation was 392 ± 19 N compared to 287 ± 16 N of the test series where the mono-bloc implants were inserted (Table 2, Figures 5-7). This difference was statistically significant (p < 0.05). In contrast, the stiffness and ultimate load were similar in both test groups with a p-value >0.05.

**Table 2:** Quantitative evaluation of the load-displacement curves with mean and standard deviation (SD). F_yield_ = yield load at 0.1 mm offset displacement; F_ult_ = ultimate expulsion load

	Stiffness in N/mm		F_yield_ in N		F_ult_ in N	
Sample	Modular	Mono-bloc	Modular	Mono-bloc	Modular	Mono-bloc
No.1	940	1,344	395	305	580	592
No.2	1,415	1,111	363	288	579	587
No.3	1,676	1,316	415	273	616	593
No.4	1,385	1,449	401	269	597	568
No.5	1,536	1,176	389	300	584	574
Mean	1,390	1,279	392	287	591	583
SD	277	135	19	16	15	11
	p = 0.44		p < 0.001		p = 0.35	

**Figure 8:**
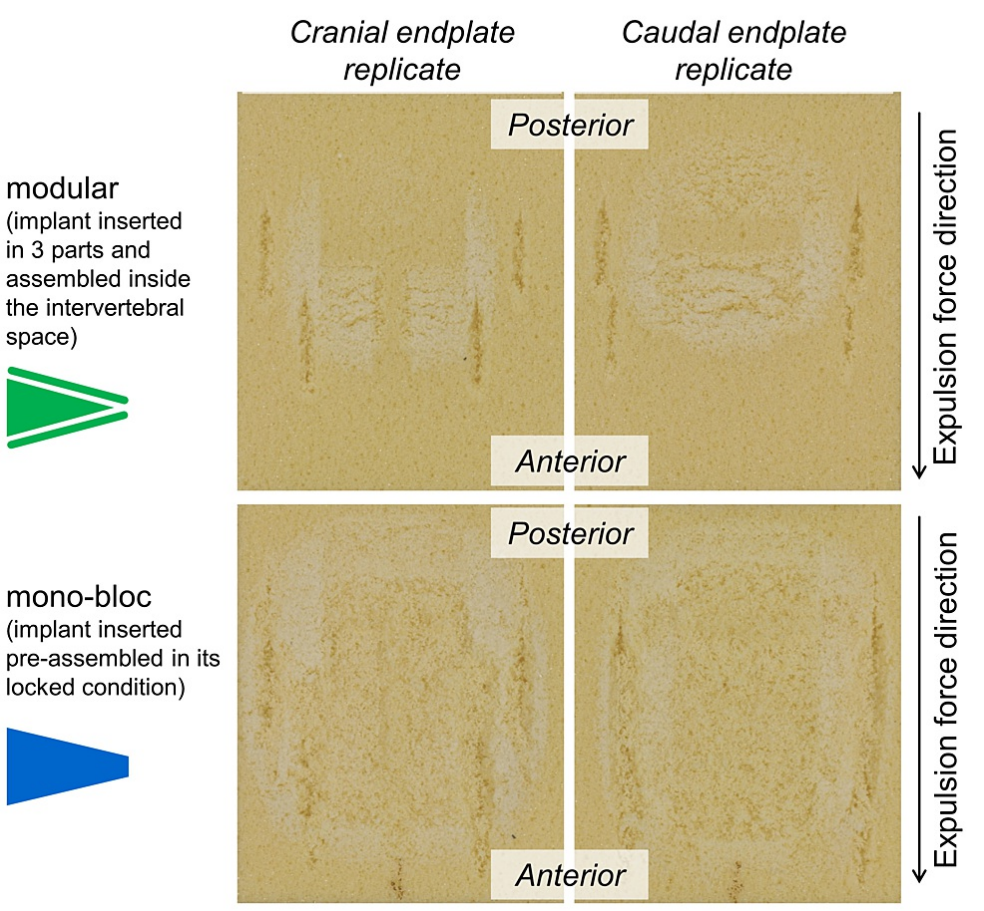
Gouging marks on the surface of the polyurethane test blocks after expulsion. In the modular test group, the gouges were smaller than in the mono-bloc group.

The gouging marks on the surface of the PU test blocks appeared longer and deeper in the mono-bloc test group (Figure 8). Because expulsion was carried out in the same way in both test groups, these differences regarding the gouging marks are attributed to cage insertion.

Overall, the load-deformation curves in each of the two test groups were very similar indicating that the sample size of n = 5 is sufficient to derive clear statements.

## Discussion

In this study, the expulsion forces were determined for a novel modular cage design, whose goal was to prevent damage to endplates during insertion. For comparison, a preassembled, mono-bloc version of the novel cage was tested as well, which was impacted into place as per the normal cage insertion method. This method caused gouging/damage to the endplates. With the ALIF cage provided, it was possible to test identical cage geometry, endplates, contact surface areas, and the number of teeth in both configurations (modular and mono-bloc). Therefore, it is very likely that obtained measurement differences are a consequence of the insertion technique.

Gouges were found on the surface of the PU test blocks after expulsion testing in both test groups. In the mono-bloc test group with the preassembled cages, these gouge marks were larger than in the modular test group. As expulsion testing was carried out in the same way in both test groups, this difference has to be attributed to the method of cage insertion. Insertion of a cage with a serrated surface causes gouging to the vertebral endplate. The gouges generated during implantation are responsible for the lower yield load measured in the group with the mono-bloc cages. The stiffness and the ultimate load were similar in both test groups. The stiffness represents the initial linear part of the load-deformation curves. In the case of the static expulsion test, this is the phase where elastic deformation occurs at the interface between the implant and the PU foam. This elastic behavior does not depend on any pre-existing gouges. In contrast, the yield load defines the point where elastic deformation steps over to initial material failure. In the case of the expulsion test, this is the point where the cage begins to move, i.e., the breakaway force. This force seems to be smaller when there are gouges on the vertebral endplate surface. Fixation between the cage and endplate is more secure when there are no gouges.

Comparative data from the literature regarding the expulsion yield load were not found and only a few values are available regarding the ultimate load. It has been previously reported that the pullout forces were influenced by the cage design. For example, Tsantrizos and coworkers found that an interbody cage with a sharp teeth design had a higher pullout resistance than other designs, such as cylindrical cages or carbon fiber saw teeth designs [9]. The average pullout forces ranged between 642 ± 176 N (for the threaded BAK cage) and 1,033 ± 291 N for the SynCage. These values are higher than those in the present study. Our test revealed an ultimate pullout force of 591 ± 15 N in the modular test group and 583 ± 11 N in the mono-bloc test group. This difference may be attributed to methodological differences in the test setup. Tsantrizos and colleagues were using cadaveric spine specimens and applied a preload of 600 N, whereas in the present study, only 500 N were applied and PU foam blocks were used as vertebral body substitutes. Moreover, in the present study, a 20° wedged cage was used, which presumably migrates more easily in the anterior direction than non-wedged or only slightly wedged cages such as those tested by Tsantrizos et al. Other comparative data, however, could not be found for anterior lumbar cages.

Static expulsion testing does not directly mimic the in vivo load case under which expulsion occurs. This in vivo load case has not yet exactly been described, but, evidently, an expulsing force will hardly occur in vivo. Instead, repetitive or single-cycle three-dimensional movements with the spine will cause the implant to migrate. This migration does not only depend on mechanical factors but also on the biological response of the surrounding tissues and/or the vertebral body bone density [16,17].

Static expulsion testing is, therefore, meant to represent an idealized and reproducible test setup that gives insight into the primary anchorage stability of the cage. This is state-of-the-art mechanical implant testing. According to the FDA Staff Class II Special Controls Guidance Document, Intervertebral Body Fusion Device, additional testing such as expulsion testing may be recommended for a given intervertebral body fusion device [18]. Because the interpretation of the results with regard to the situation in vivo is difficult, testing is often carried out as comparative testing similar to the present study.

The PU blocks used in this study were simplified with a planar surface and a standardized density. As mentioned above, the density of these blocks was chosen according to ASTM F2267 [14]. Additionally, a comparison between the human vertebral endplates and the Sawbones solid rigid PU foam was carried out regarding material strength, which is the most important parameter with respect to material damage. The results showed that grade 15 PU foam has a compressive strength slightly below that of the human and a shear strength slightly above that of the human (Table 3). Overall, grade 15 PU foam seems to be a well-suited choice for expulsion testing.

**Table 3:** Strength of solid rigid PU foam in different densities (Sawbones) and human endplates

Material	Compressive strength	Shear strength
PU 10 pcf	2.2 MPa	1.6 MPa
PU 15 pcf	4.9 MPa	2.8 MPa
PU 20 pcf	8.4 MPa	4.3 MPa
PU 40 pcf	31.0 MPa	11.0 MPa
Human vertebral endplates	6.3 MPa (at 10 mm/s) at L4 and L5 [19] ≥8.7 MPa depending on endplate region [20]	0.5 MPa (anterior) to 2.7 (lateral) at T10 [21]

The amplitude of 500 N for the axial compressive load was chosen according to ASTM F2077 [15]. This load corresponds well with the scientific data. Based on intradiscal pressure measurements, Wilke and Rohlmann 2012 could show that most lying postures are associated with an axial force <500 N [22]. Approximately 500 N are reached during relaxed standing or sitting and 500 N are at the lower border of the force ranges reported for walking and jogging. The values reported by Nachemson tend to be higher than those reported by Wilke and Rohlmann [23]. Thus, 500 N seem to be a realistic worst case for expulsion testing because higher loads would more and more prevent the cage from migration.

Nevertheless, we believe that the data reported in this paper can support and improve the current understanding of the biomechanical characteristics of anterior lumbar fusion cages, and this data could also foster further implant-related research.

## Conclusions

This study provides a small but important contribution to a new idea on how to improve the primary anchorage of ALIF cages. The results showed that gouges on the surface of the endplate caused during cage insertion may decrease the primary anchorage of the cage on the vertebral endplates and, thus, increase the risk of cage migration.
